# Longitudinal observation of persistent Long COVID symptoms and health-related quality of life in an adult German cohort

**DOI:** 10.1186/s12879-026-14252-z

**Published:** 2026-08-19

**Authors:** Marie Mikuteit, Dominik Schröder, Jacqueline Niewolik, Frank Klawonn, Chantal Degen, Frank Müller, Georg M. N. Behrens, Alexandra Dopfer-Jablonka, Sandra Steffens

**Affiliations:** 1 Department of Dermatology and Allergy, Hannover Medical School, Carl-Neuberg-Str. 1, 30625 Hannover, Germany; 2 Departement of Medical Education, Hannover Medical School, Hannover, Germany; 3 Department of General Practice, University Medical Center Göttingen, Göttingen, Germany; 4 Institute for General Practice and Palliative Care, Hannover Medical School, Hannover, Germany; 5 Institute for Information Engineering, Ostfalia University of Applied Sciences, Wolfenbüttel, Germany; 6 Department of Otorhinolaryngology, Paracelsus Medical University, Nuremberg, Germany; 7 Department of Family Medicine, College of Human Medicine, Michigan State University, Grand Rapids, MI USA; 8 Department of Rheumatology and Immunology, Hanover Medical School, Hannover, Germany; 9 German Center for Infection Research (DZIF), Partner Site Hannover- Braunschweig, Hannover, Germany

**Keywords:** Long COVID symptoms, SARS-CoV-2, Longitudinal observation, Post-covid syndrome

## Abstract

**Background:**

Many individuals experience persistent symptoms following an acute COVID-19 infection, a condition known as Long COVID. The aim of this study was to analyse the prevalence, intensity and longitudinal trajectories of different Long COVID symptoms at baseline and at follow-ups up to 60 weeks later in a German cohort.

**Methods:**

This longitudinal observational study is part of the DEFEAT Corona project, wherein Long COVID subjects completed online questionnaires on symptom severity (0–10 points) and health-related quality of life (hrQoL), measured with EQ5D-VAS, at three time points. At baseline, sociodemographic information, COVID-19 infection characteristics, and COVID-19 vaccination status were also recorded. Symptom occurrence and intensity was compared between baseline and two follow-up surveys 54 to 60 weeks later.

**Results:**

Among *n* = 262 adult participants (84.4% female, 14.8% male and 0.8% diverse, mean age 44.85 ± 11.48 years), the most frequent symptoms at baseline were fatigue (67.2%, mean severity 9.02 ± 0.89), palpitations (61.1%, mean severity 6.58 ± 1.95), and concentration impairment (61.0%, mean severity 8.55 ± 1.12). The prevalence decreased in 3/27 Long COVID symptoms and symptom intensity decreased in 15/27 symptoms with small changes in severity (between 0.6 and 2 points of max. 10 points). HrQoL decreased significantly over time (*p* = 0.006). However, the mean change of − 4.31 ± 21.2 points remained below the established MCID of 7.5 points, indicating no clinically meaningful deterioration.

**Conclusion:**

More than a year after the acute infection, Long COVID patients of the DEFEAT Corona cohort describe symptoms as persistent and intense. With the corresponding impairment in hrQoL, this underlines the urgent need for comprehensive therapies for Long COVID.

**Trial registration:**

The study was in the German Clinical Trial Registry (DRKS00026007) on 9th Sep 2021.

**Supplementary Information:**

The online version contains supplementary material available at 10.1186/s12879-026-14252-z.

## Background

While the COVID-19 pandemic has transitioned to an endemic stage with vaccines providing good protection against hospitalization and death, prolonged courses of COVID-19 have become increasingly important. The World Health Organization (WHO) defines ongoing symptoms, lasting longer than 4 weeks as Long COVID or post-acute sequelae of COVID (PASC) [1]. Research indicated at varying incidence rate of Long COVID, ranging from 5% to 76%, depending on the used study methodology, disease criteria, and the characteristics of the studied cohort [2, 3]. The underlying pathomechanism of Long COVID remain unclear, despite proposed theories implicating viral persistence and immune system dysregulation as potential contributors to this complex condition [4]. The symptoms of Long COVID are numerous and vary among patients, with fatigue, brain fog, shortness of breath, palpitations and muscle pain among most common symptoms [4–6]. There are also differences within the virus variants: The Omicron variant caused fewer protracted courses of disease compared to the Delta variant, but with higher infection rates, the number of protracted COVID cases continues to increase [6]. Furthermore, several factors, such as female gender, hospitalization or higher age, have been proposed risk factors for the development of Long COVID [7, 8].

Long COVID patients experience considerable impairments in their everyday life impacting both social participation and quality of life [9, 10]. A meta-analysis reported a prevalence of 7.8% to 17% for Long COVID symptoms more than 12 weeks after the infection in a population studied in the UK [7]. Other studies report a higher prevalence [11]. Thus, Long COVID can be considered as an important challenge to patients and healthcare systems alike [12]. Longitudinal studies suggest considerable heterogeneity in HrQoL trajectories following COVID-19, with a substantial proportion of patients showing persistently reduced quality of life over more than one year after SARS-CoV-2 infection [13].

The DEFEAT Corona study is a prospective observational study assessing the long-term consequences of SARS-CoV-2 infections [14]. At the time of study design, longitudinal data using multi-dimensional, self-reported measures to simultaneously track symptom progression and hrQoL in Long COVID patients remained scarce, underscoring the rationale for the present study design [15]. The present analysis aimed to longitudinally track the prevalence, intensity, and trajectories of Long COVID symptoms over up to 60 weeks. Our hypothesis was that the prevalence and severity of Long COVID symptoms decrease over time.

## Methods

### Study design and setting

This study was conducted as a secondary data analysis of the DEFEAT Corona study [14], a joint research project of Hanover Medical School (MHH), University Medical Center Göttingen (UMG), and Ostfalia University of Applied Sciences, aiming to assess Long COVID. The study received approval through the institutional review boards of MHH (Nr. 9948_BO_K_2021) and UMG (29/3/21) and was registered at the German Clinical Trial Registry (DRKS00026007) on September 9th, 2021 before data collection began.

The study’s catchment area was primarily focused on the German Federal State of Lower Saxony, where information about the study was disseminated through multiple channels. Study participation was enabled through a self-selecting online recruitment process via the platform www.defeat-corona.de. Information about the study was distributed through specialized outpatient clinics at MHH and UMG, local public health authorities in Lower Saxony, regional primary care clinics as well as Long COVID support and self-help groups on social media platforms. Data collection took place between September 2021 and November 2023, with baseline data collected until December 2022 and follow-up surveys conducted until November 2023. Surveys were administered using the online survey platform SoSciSurvey (SoSci Survey GmbH, www.soscisurvey.de).

### Participants

Inclusion criteria were (a) participant age ≥ 18 years, (b) German language proficiency, (c) consent for participating and (d) information on Long COVID status. Persons who did not consent through the online platform, or with a SARS-CoV-2 infection < 4 weeks prior to baseline survey were excluded. We only included people in the study who reported a PCR, antigen, or antibody test-confirmed acute SARS-CoV-2 infection. Participants were considered to have Long COVID if they reported one or more ongoing or new symptoms at least four weeks after their initial COVID-19 infection. This analysis included individuals who met these criteria based on self-reported symptoms and who completed surveys at all three designated time points, resulting in a complete case analysis. Missing data were not imputed. Of the 347 participants completing all three surveys, 85 were subsequently excluded: 31 self-reported resolution of Long COVID symptoms and 54 provided insufficient information on Long COVID status, resulting in a final analytic sample of 262 participants.

### Variables and measurements

We consider gender as a social construct with a broad spectrum between “male” and “female”. For statistical simplification gender was assessed by asking participants about their gender identity providing the options female, male, diverse gender.

Full vaccination was considered if participants reported to have received at least two reported immunizations with any EMA approved COVID-19 vaccine. We considered all participants with no information on vaccination status and a COVID-19 infection prior December 1^rst^ 2021 as unvaccinated, since no COVID-19 vaccines were approved and available in Germany before this date.

We assessed the subjective severity of 27 symptoms, based on the World Health Organization Case Report Form [16] and the UK Long COVID guideline [17], on a 11-point Likert scale with 0 being no symptom at all and 10 the most severe. A symptom was considered present in a participant if its reported value was greater than or equal to the median value of the cohort for that specific symptom, calculated separately at each of the three timepoints (or a value of 1 if the median was below 1).

We calculated the Post COVID Score (PCS) according to Bahmer et al. [18], with severe Long COVID being defined as PCS of 26.25 or higher. As the original PCS was developed using binary symptom assessment, we defined symptom presence as a Likert scale rating of ≥ 1 (i.e., any symptom rated above zero), representing the most conservative threshold to avoid underestimating symptom burden. The 12 PCS dimensions were derived from the following symptoms: (1) sensory (smell/taste), (2) fatigue, (3) exercise intolerance (dyspnoea, reduced physical capacity), (4) rheumatological (joint pain, muscle pain), (5) ENT (throat symptoms), (6) cough, (7) chest pain, (8) gastrointestinal (diarrhoea, nausea, abdominal pain), (9) neurological (concentration impairment, headache, dizziness, paraesthesia, delirium), (10) dermatological (rash, hair loss), (11) infection susceptibility (fever, increased infection rate), and (12) sleep disturbance. The threshold of 26.25 was established by Bahmer et al. via cluster analysis in the COVIDOM cohort (*n* = 667), distinguishing mild/no (≤ 10.75), moderate (> 10.75–26.25), and severe (> 26.25) PCS, and was validated in two independent sub-cohorts [18].

Health-related quality of life was measured using the EQ5D Visual Analogue Scale (EQ5D-VAS), a validated instrument where participants rate their current health state on a scale from 0 (worst imaginable health) to 100 (best imaginable health), with higher scores indicating better health-related quality of life [19].

Social participation was assessed using the Index for the Measurement of Restrictions in Participation (IMET), a validated German instrument that measures impairment in nine different areas of life based on the international classification of functioning (e.g., work, family, leisure) on a scale from 0 (no impairment) to 10 (no activity possible). The total score ranges from 0 to 90, with higher scores indicating greater impairment in social participation [20].

We grouped hypertension, heart failure, coronary heart disease, atrial fibrillation, cardiac arrhythmia, and peripheral arterial disease as cardiovascular conditions; and diabetes mellitus (type 1 and 2), gout, biliary diseases, severe obesity, and thyroid diseases as metabolic conditions; Asthma and chronic obstructive pulmonary diseases as pneumological conditions; hepatitis, HIV, inflammatory bowel disease, psoriasis, rheumatologic conditions, polymyalgia rheumatica, atopic dermatitis, and other autoimmune conditions as inflammatory and autoimmune conditions; Dementia, depression, schizophrenia, migraine, epilepsy and Parkinson’s disease as neuropsychiatric conditions. These groupings were defined according to clinical expertise and reflect established medical subspecialty categories; heterogeneity within categories, particularly for neuropsychiatric and inflammatory/autoimmune conditions, is acknowledged as a limitation.

### Sample size calculation

With a power of 80% and a significance level (α) of 0.05, a reduction in symptom severity by one point on a Likert scale (standard deviation of 2.5) can be detected with a sample size of 52 participants. Similarly, to detect a 10% decrease in the prevalence of a symptom, a sample size of 146 individuals would be required.

### Quantitative variables, bias and statistics

Data was stored on local servers at MHH according to General Data Protection Regulation. For statistical analysis, participants who did not report their Long COVID status at follow-up and did not rate any of the 27 possible Long COVID symptoms at baseline were excluded. Data management and statistical analysis were performed with R (version 4.2) and the packages tidyverse (version 1.3.1), lubridate (version 1.8.0.), stats (version 4.1.1), epitools (version 0.5–10.1), NSM3 (version 1.18), DescTool (version 0.99.54), pwr (version 1.3-0), grid (version4.2.3) and gridExtra (version 2.3) were used. To describe participant characteristics, we used absolute numbers and proportions for categorical data and median with corresponding interquartile ranges (IQR) or mean with standard deviation (SD) for continuous data. In order to evaluate bias, an inductive comparison with the rest of the cohort from baseline, that were not included in this analysis, was conducted using Mann-Whitney-*U* test. A Long COVID symptom was considered present if it was rated with a symptom severity greater or equal to the median on a 0–10 scale. Mean and standard deviation (SD) of the symptom strength was thus calculated for those participants who had reported that symptom. To assess the statistical significance of changes in symptom severity across the three time points, we employed the Skillings-Mack test and Wilcoxon Rank post-hoc tests. The simulated p-value for the Skillings-Mack test was based on 10,000 Monte Carlo resamples. For comparing the occurrence rates of symptoms, we utilized Cochran’s *Q* test with McNemar aspost-hoc tests. To assess whether the wave-specific median cutoff influenced the observed prevalence trajectories, we conducted a sensitivity analysis in which the cutoff was fixed once at the baseline (T1) sample median (or a value of 1 if the median was below 1) and applied unchanged across all three timepoints. Prevalence was compared using Cochran’s Q test with Holm correction, identical to the primary analysis (Supplementary Tables 2 and 3). Correlation analysis of number or intensity of Long COVID symptoms and hrQoL was conducted using Spearman’s rho. A linear mixed model was fitted with EQ5D-VAS as outcome, symptom count, mean symptom severity, and timepoint (baseline, first and second follow-up) as fixed effects, and a random intercept per participant. Statistical significance was determined as *p* < 0.05, if not otherwise stated. For both the number of symptom counts and symptom severity, we applied the Bonferroni-Holm correction to adjust the p-values for multiple comparisons. In this study, missing values for specific variables were excluded on a per-variable basis, without excluding the entire case from the rest of the analysis.

## Results

### Cohort characteristics

**Fig. 1:**
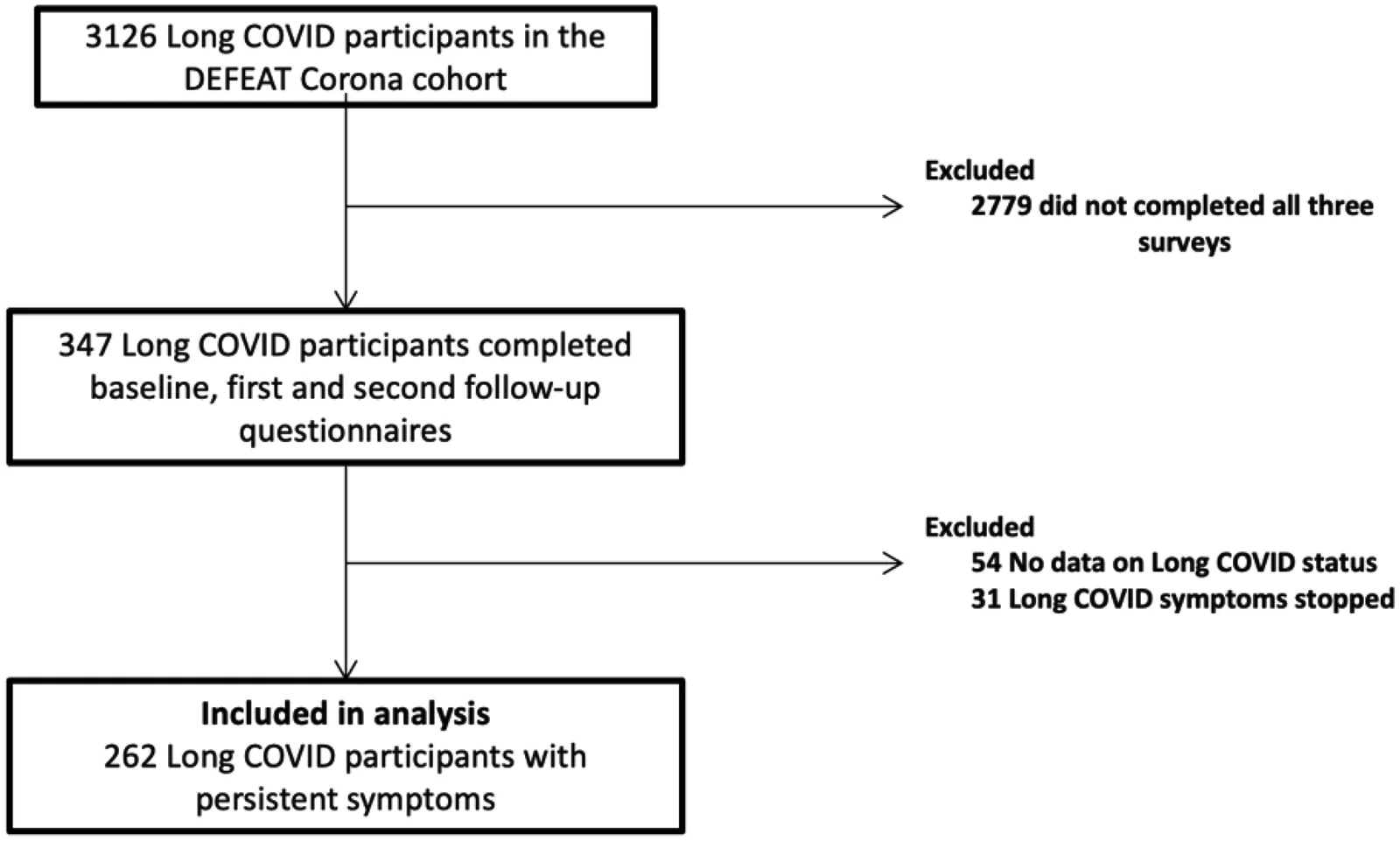
Study flow chart. Baseline data were collected in September 2021, the first and second follow-up questionnaires were sent out to the participant at least 4 and 12 weeks after the first questionnaire

A total of 262 participants who met the criteria for Long COVID at baseline completed both follow-up questionnaires and were included in the analysis (Fig. 1).

The participants were mostly of female gender (84.4%) and were on average 44.85 (SD 11.48) years old. 10.3% of participants were hospitalized during their acute SARS-CoV-2 infection (see Table 1). The mean time intervals between the initial infection and subsequent follow-up surveys were as follows: 41.76 weeks (SD 25.10) to the baseline, an additional 8.68 weeks (SD 6.67) to the first follow-up, and a further 32.9 weeks (SD 14.6) to the second follow-up. Compared to participants who only completed baseline (*n* = 2,930), those who completed all questionnaires (*n* = 262) were slightly older [44.85 (SD 11.48) vs. 43.14 (SD 12.54) years, *p* = 0.022], more likely to be infected early in the pandemic, had higher hospitalization rates (10.3% vs. 5.7%, *p* = 0.004), and lower pre-infection vaccination rates (34.0% vs. 56.5%, *p* < 0.001). They also reported more Long COVID symptoms [16.9 (SD 6.25) vs. 15.1 (SD 6.75), *p* < 0.001], greater symptom burden [3.57 (SD 1.72) vs. 3.27 (SD 1.79), *p* = 0.005], lower hrQoL [46.57 (SD 19.90) vs. 52.89 (SD 22.36), *p* < 0.001], and higher social participation impairment [43.27 (SD 18.62) vs. 37.97 (SD 22.47), *p* < 0.001].

**Table 1:** Characteristics of study participants

	Overall
**n**	262
**Age**,** mean (SD)**	44.85 (11.48)
**Gender**,** n (%)**	
Female	216 (84.4)
Male	38 (14.8)
Gender diverse	2 (0.8)
**Period between infection and baseline survey (weeks)**,** mean (SD)**	41.76 (25.10)
**Period between baseline and first follow up (weeks)**,** mean (SD)**	8.68 (6.67)
**Period first and secondfollow up (weeks)**,** mean (SD)**	32.9 (14.6)
**Time of initial COVID-19 infection**,** n (%)**	
before 06/31/2021	169 (64.8)
07/01/2021–12/31/2021	37 (14.2)
After 01/01/2022	55 (21.1)
**Hospitalization**	
Normal ward, n (%)	27 (10.3)
Intensive care unit, n (%)	11 (4.2)
**Comorbidities (multiple selection permitted)**	
Cardiovascular, n (%)	56 (21.4)
Metabolic, n (%)	80 (30.5)
Pneumological, n (%)	45 (17.2)
Autoimmune or Chronic Inflammatory (%)	41 (15.6)
Neuropsychiatric, n (%)	64 (24.4)
Cancer, n (%)	10 (3.8)
**Education**	
< 12 years of schooling, n (%)	106 (41.2)
≥ 12 years of schooling, n (%)	151 (58.8)
**Completed COVID-19 vaccination**, n (%)	191 (72.9)
**COVID-19 vaccination completed before infection**, n (%)	71 (34.0)

SD = standard deviation, n = number of occurrences

Missing values: age n=5, gender n=6, time of infection n=1, education n=5, vaccination status n=53

### Long COVID symptoms over time

Most common Long COVID symptoms at baseline (%) were fatigue (67.2), palpitations (61.1), and concentration impairment (61.0) (Median cut offs are displayed in Supplement 1).

**Fig. 2:**
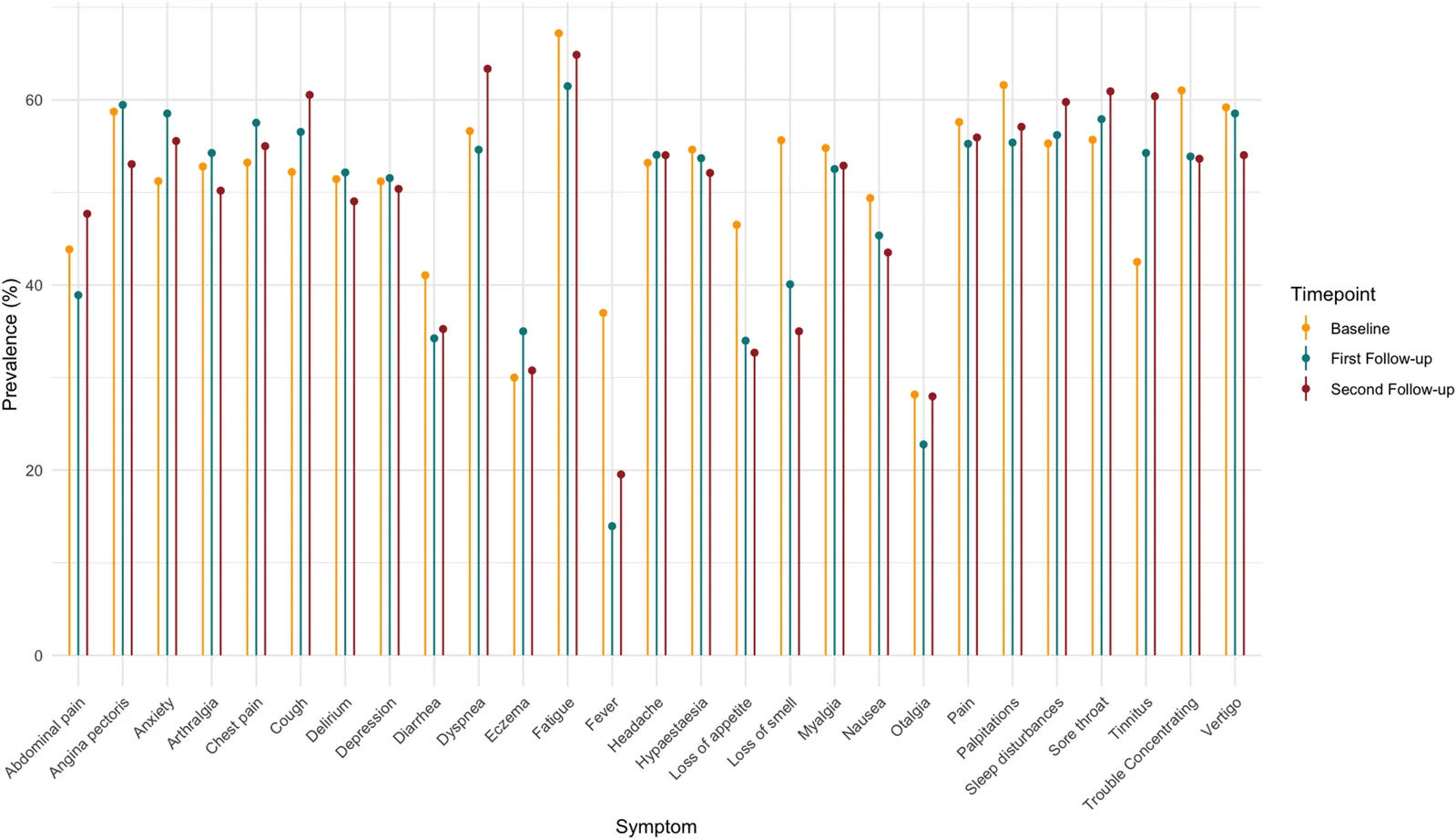
Prevalence of long COVID symptoms at baseline and follow-up timepoints. Symptoms were considered present when rated with severity ≥ median severity at each timepoint. Fever, loss of appetite, and loss of smell were significantly less frequent at second follow-up compared to baseline, while tinnitus prevalence increased

For most symptoms, there was no change in number of occurrences over time. Fever, loss of appetite, and loss of smell occurred statistically significantly less frequently at the first follow-up compared to baseline, while tinnitus became more frequent over the two follow-ups (Fig. 2). Pairwise comparisons confirmed this pattern: the same symptoms differed significantly between baseline and the second follow-up, whereas no symptom showed a significant change between the first and second follow-up (Supplement 2). This suggests that most of the symptom trajectory occurred within the first observation period. In the sensitivity analysis using a cutoff fixed at the baseline median, results were directionally consistent with the primary analysis, and no previously significant change was reversed. However, prevalence changes reached statistical significance for 12 of 27 symptoms instead of 4: angina pectoris, chest pain, delirium, dyspnoea, fatigue, palpitations, trouble concentrating, and vertigo additionally showed significant reductions (Supplement 3). This indicates that the wave-specific median cutoff, which is recalculated at each timepoint and therefore shifts with the sample distribution, may mask true reductions in symptom prevalence over time.

**Fig. 3:**
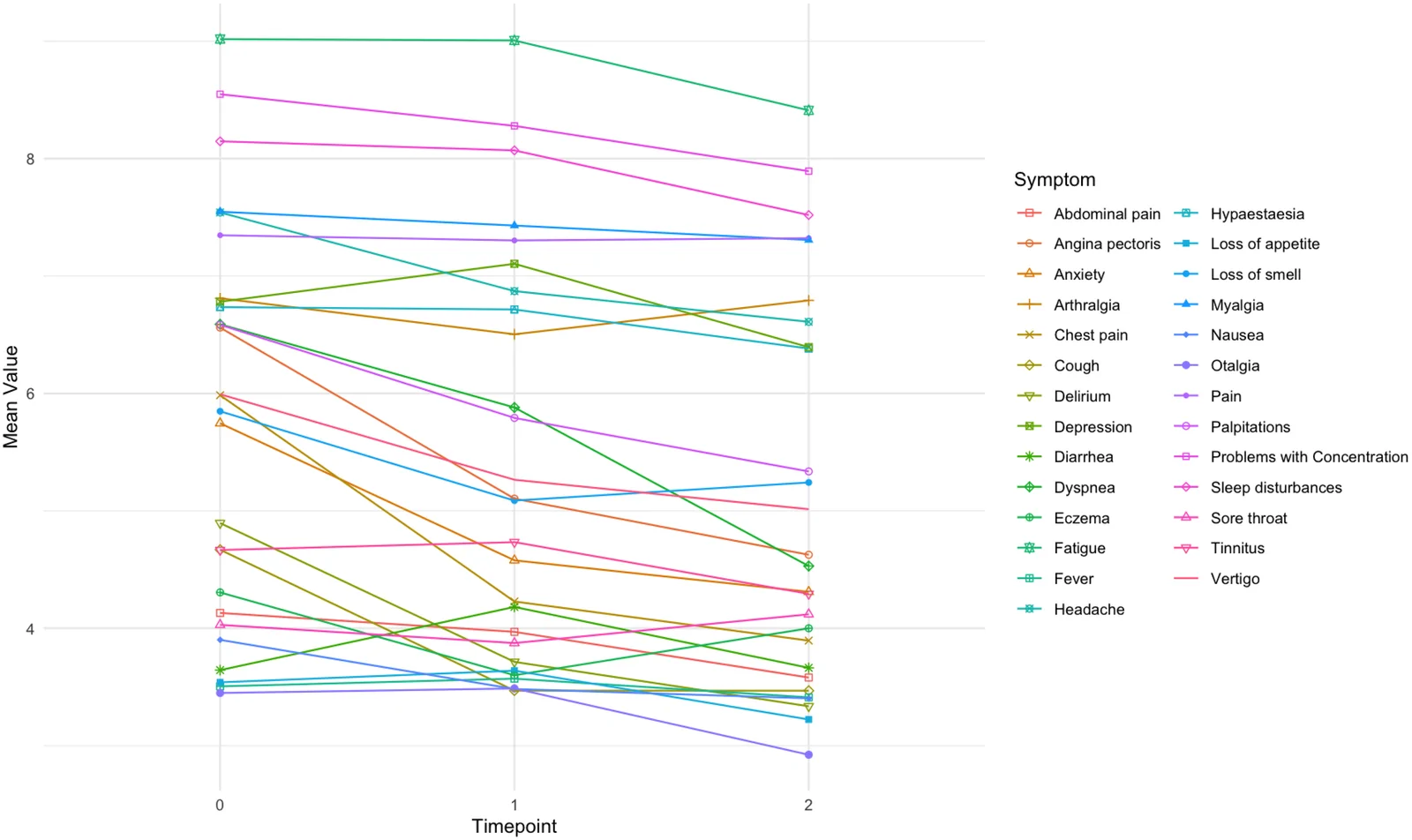
Mean severity of Long COVID Symptoms across time points. Mean symptom severity (scale 0–10) at baseline, first and second follow-up, calculated among participants reporting the symptom (severity ≥ cohort median). Note that this represents severity among affected individuals rather than the full sample mean

The most severe symptoms at baseline [mean (SD)] were fatigue [9.02 (0.89)], concentration impairment (8.55 [1.12]), sleep disturbances (8.15 [1.37]). Most symptoms became significantly less severe over time (15 of 27 symptoms, Table 2; Fig. 3). Pairwise comparisons of symptom severity confirmed that most significant reductions occurred between baseline and second follow-up (14 of 27 symptoms), while changes between first and second follow-up were rarely significant (5 of 27 symptoms; Supplement 4). Notably, fatigue severity showed a significant decrease only in the later observation period (first vs. second follow-up, *p* > 0.001), as did depression severity (*p* = 0.002).

The mean Post COVID Score (PCS) was 40.2 (SD 14.5) points at baseline, 41.0 (SD 12.8) at first follow-up, and 42.3 (SD 13.5)at second follow-up. This reflected a consistently high proportion of participants with severe Long COVID across all three timepoints (83.3%, 85.0%, and 88.2%), with significant differences between timepoints (*p* = 0.018) Internal consistency of the PCS was acceptable across all three timepoints (Cronbach’s α = 0.71 at baseline, 0.63 at first follow-up, and 0.68 at second follow-up).

### Health-related quality of life

**Fig. 4:**
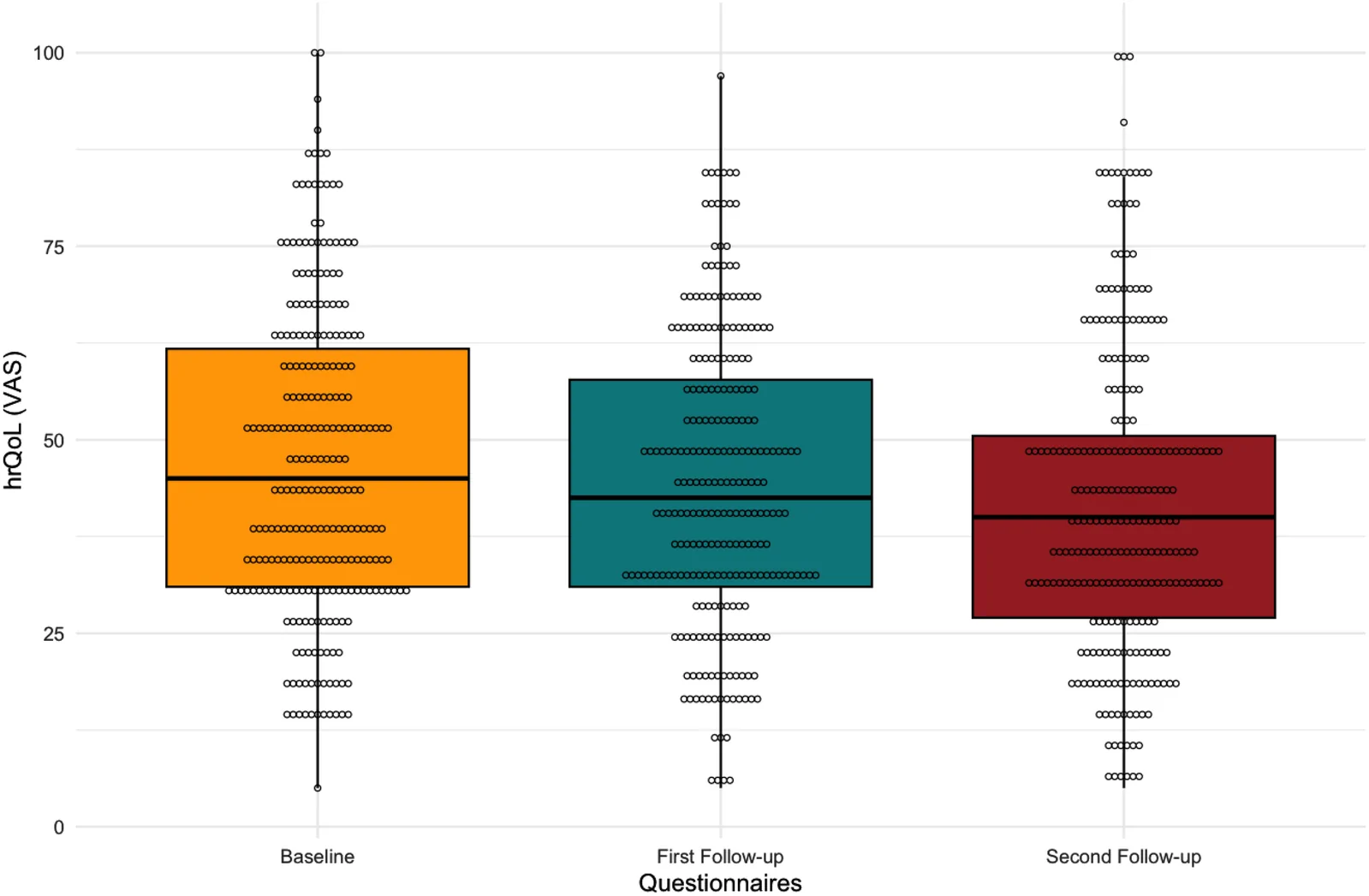
Health-related quality of life over time. hrQol = health-related quality of life; VAS = visual analogue scale (EQ5D-VAS, range 0-100, higher scores indicate better hrQoL). Skillings-Mack test *p* = 0.006, post hoc tests (Wilcoxon Test) baseline vs. first follow-up *p* = 0.028; baseline vs. second follow-up *p* < 0.001; first vs. second follow-up *p* = 0.028

EQ-5D 100-point-Visual-Analogue-Scale (VAS) scores [mean (SD)] differed significantly and declined from one timepoint to the next [baseline 46.57 (SD 19.9) vs. first follow-up 44.31 (18.7) vs. second follow-up 41.9 (20.4), Skillings-Mack Test *p* = 0.006, see Fig. 4]. The mean difference in VAS between baseline and the second follow-up was − 4.31 points (SD 21.2) and did thus not exceed the established miminal clinically important difference (MCID) of 7.5 points for the EQ5D-VAS [21]. There was no significant difference in impairment of social participation measured with IMET between the baseline and second follow-up [mean (SD): 43.27 (18.62) vs. 44.18 (20.55), Wilcoxon Rank test, *p* = 0.478; see Fig. 4].

**Fig. 5:**
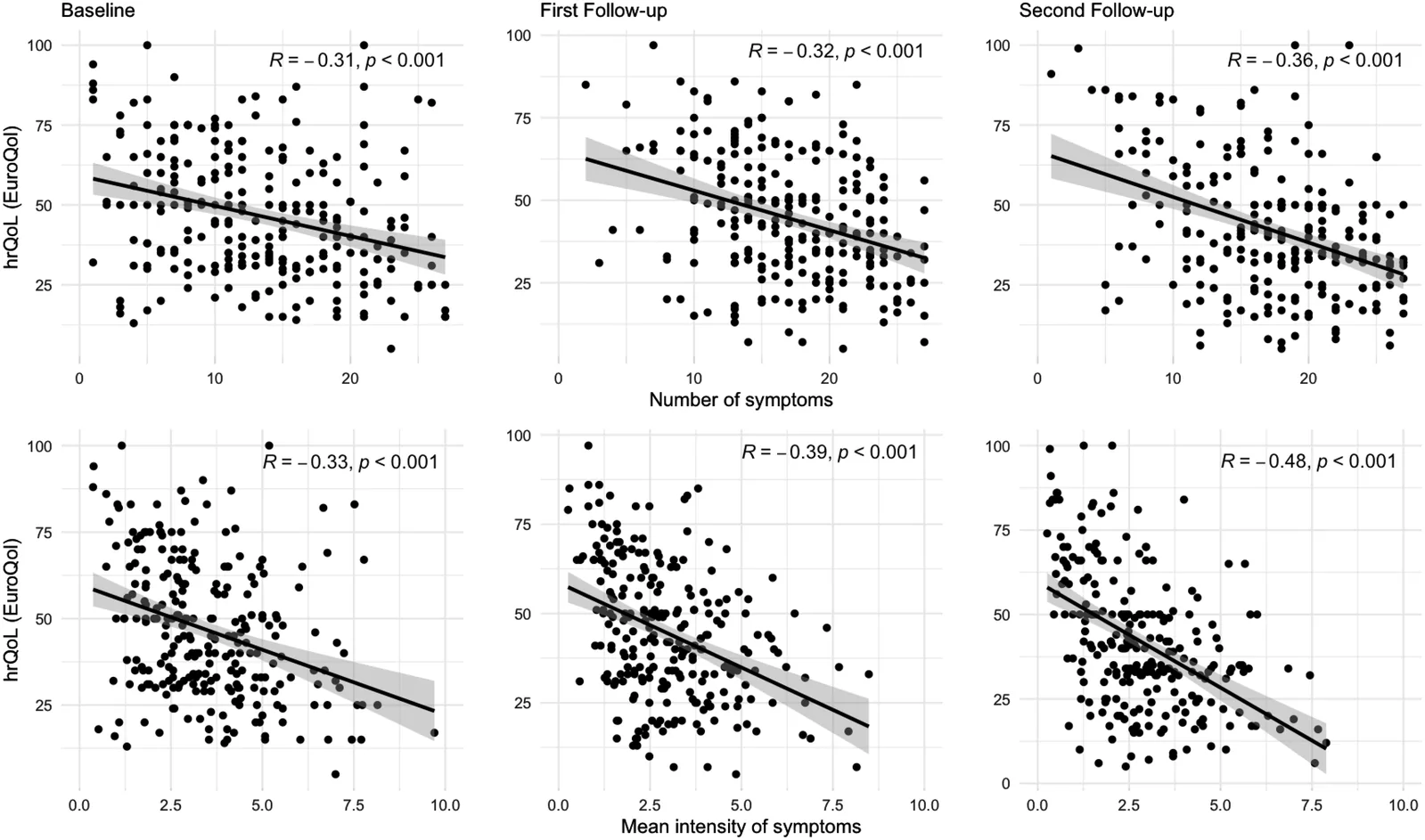
Correlation of hrQoL with symptom count and mean symptom severity across timepoints. Spearman correlation coefficient (R) and p-value was calculated for baseline and both follow-up assessments. Variables analysed: long COVID symptom count per participant (range 0–27), mean symptom Intensity (range 0–10), and hrQoL (EQ5D-VAS, range 0-100)

The EQ5D-VAS score showed negative correlations with both symptom count and mean symptom intensity across all timepoints (*r* = -0.31 to -0.48; Fig. 5). In the linear mixed model, mean symptom severity was significantly associated with lower HrQoL (β = −3.92, 95% CI − 5.32; −2.52, *p* < 0.001), while symptom count was not independently associated (β = −0.19, *p* = 0.341). HrQoL declined significantly at first (β = −3.51, 95% CI − 6.68; −0.33, *p* = 0.031) and second follow-up (β = −6.34, 95% CI − 9.71; −2.97, *p* < 0.001) compared to baseline (Supplement 5).

**Table 2:** Changes in symptoms of participants with Long COVID over all time points (*N* = 262)

	Baseline			First Follow-Up			Second Follow-Up				
	Prevalence, *n* (%)	NA, *n* (%)	Severity, mean (SD)^1^	Prevalence, *n* (%)	NA, *n* (%)	Severity, mean (SD)^1^	Prevalence, *n* (%)	NA, *n* (%)	Severity, mean (SD)^1^	*P* value Prevalence^2^	*p* value severity^3^
Abdominal pain	107 (43.9)	18 (6.9)	4.13 (2.67)	99 (38.5)	4 (1.5)	3.97 (2.65)	124 (47.7)	2 (0.8)	3.58 (2.66)	0.586	1.0
Angina pectoris	148 (58.7)	10 (3.8)	6.56 (1.73)	154 (59.5)	3 (1.1)	5.10 (2.47)	139 (53.1)	0 (0.0)	4.63 (2.33)	1.000	< 0.001
Anxiety	126 (51.2)	16 (6.1)	5.75 (2.61)	152 (58.9)	4 (1.5)	4.58 (2.94)	145 (55.6)	1 (0.4)	4.31 (3.02)	1.000	0.003
Arthralgia	132 (52.8)	12 (4.6)	6.81 (1.96)	139 (53.9)	4 (1.5)	6.50 (2.09)	130 (50.2)	3 (1.1)	6.79 (1.89)	1.000	1.0
Chest pain	132 (53.2)	14 (5.3)	5.98 (2.10)	149 (57.5)	3 (1.1)	4.23 (2.58)	143 (55.0)	2 (0.8)	3.90 (2.57)	1.000	< 0.001
Cough	130 (52.2)	13 (5.0)	4.67 (2.37)	147 (56.5)	2 (0.8)	3.47 (2.41)	158 (60.5)	1 (0.4)	3.47 (2.47)	1.000	0.058
Delirium	125 (51.4)	19 (7.3)	4.90 (2.35)	133 (52.2)	5 (1.9)	3.71 (2.54)	128 (49.0)	1 (0.4)	3.34 (2.47)	1.000	0.003
Depression	128 (51.2)	12 (4.6)	6.78 (1.80)	134 (51.9)	4 (1.5)	7.10 (1.82)	132 (50.4)	0 (0.0)	6.39 (2.23)	1.000	0.003
Diarrhea	101 (41.1)	16 (6.1)	3.64 (2.66)	88 (34.2)	5 (1.9)	4.18 (2.74)	92 (35.2)	1 (0.4)	3.66 (2.51)	1.000	1.0
Dyspnea	141 (56.6)	13 (5.0)	6.59(1.66)	142 (54.6)	2 (0.8)	5.88 (1.85)	166 (63.4)	0 (0.0)	4.53 (2.29)	0.586	0.003
Eczema	72 (30.0)	22 (8.4)	4.31 (2.83)	90 (34.6)	2 (0.8)	3.60 (2.83)	80 (30.8)	2 (0.8)	4.00 (2.60)	1.000	1.0
Fatigue	170 (67.2)	9 (3.4)	9.02 (0.89)	158 (61.5)	5 (1.9)	9.01 (0.86)	168 (64.9)	3 (1.1)	8.41 (1.08)	1.000	0.003
Fever	91 (37.0)	16 (6.1)	3.51 (2.64)	35 (13.6)	4 (1.5)	3.57 (2.69)	51 (19.5)	1 (0.4)	3.41 (2.42)	< 0.001	< 0.001
Headache	133 (53.2)	12 (4.6)	7.54 (1.64)	140 (54.1)	3 (1.1)	6.87 (1.77)	141 (54.0)	1 (0.4)	6.61 (1.89)	1.000	0.003
Hypaestaesia	136 (54.6)	13 (5.0)	6.74 (2.22)	137 (53.3)	3 (1.1)	6.72 (2.21)	136 (52.1)	1 (0.4)	6.38 (2.22)	1.000	1.0
Loss of appetite	113 (46.5)	19 (7.3)	3.54 (2.63)	86 (33.6)	6 (2.3)	3.64 (2.64)	85 (32.7)	2 (0.8)	3.22 (2.56)	< 0.001	0.003
Loss of smell	138 (55.6)	14 (5.3)	5.85 (3.44)	103 (40.1)	5 (1.9)	5.09 (3.29)	91 (35.0)	2 (0.8)	5.24 (3.21)	< 0.001	0.003
Myalgia	137 (54.8)	12 (4.6)	7.55 (1.60)	135 (52.5)	5 (1.9)	7.43 (1.62)	137 (52.9)	3 (1.1)	7.31 (1.68)	1.000	1.0
Nausea	122 (49.4)	15 (5.7)	3.90 (2.79)	116 (45.0)	7 (2.7)	3.48 (2.53)	114 (43.5)	0 (0.0)	3.40 (2.55)	1.000	0.606
Otalgia	69 (28.2)	17 (6.5)	3.45 (2.81)	59 (22.8)	3 (1.1)	3.54 (2.55)	73 (28.0)	1 (0.4)	3.08 (2.20)	1.000	1.0
Pain	144 (57.6)	12 (4.6)	7.35 (1.64)	142 (55.3)	5 (1.9)	7.30 (1.62)	146 (55.9)	1 (0.4)	7.32 (1.60)	1.000	1.0
Palpitations	154 (61.6)	12 (4.6)	6.58 (1.95)	144 (55.4)	2 (0.8)	5.79 (1.95)	149 (57.1)	1 (0.4)	5.34 (2.12)	1.000	1.0
Sleep disturbances	136 (55.3)	16 (6.1)	8.15 (1.37)	129 (50.0)	4 (1.5)	8.07 (1.37)	156 (59.8)	1 (0.4)	7.52 (1.78)	1.000	< 0.001
Sore throat	137 (55.7)	16 (6.1)	4.03 (2.63)	151 (58.3)	3 (1.1)	3.87 (2.52)	159 (60.9)	1 (0.4)	4.12 (2.67)	1.000	0.208
Tinnitus	102 (42.5)	22 (8.4)	4.67 (3.08)	139 (53.9)	4 (1.5)	4.73 (2.99)	157 (60.4)	2 (0.8)	4.29 (3.07)	< 0.001	0.003
Trouble concentrating	155 (61.0)	8 (3.1)	8.55 (1.12)	140 (54.3)	4 (1.5)	8.28 (1.00)	140 (53.6)	1 (0.4)	7.89 (1.26)	0.612	< 0.001
Vertigo	148 (59.2)	12 (4.6)	5.99 (2.25)	151 (58.5)	4 (1.5)	5.26 (2.48)	141 (54.0)	1 (0.4)	5.01 (2.30)	1.000	< 0.001

NA = Not answered

^1^Mean and SD of symptom severity (rated from 0-10) of participants with symptoms

^2^ Cochrane Q, ^3^ Test Skillings-Mack Test; all p-values were adjusted for multiple testing with Bonferroni Holm method. Post-hoc tests are to be found in the supplements

## Discussion

The analysis of 262 Long COVID patients followed across three timepoints revealed modest improvements over time: prevalence decreased in 4 of 27 symptoms, while severity decreased significantly in 16 of 27 symptoms. With a cut-off fixed at baseline, more symptoms decreased significantly in prevalence in the group (12/27). However, these severity reductions were small in magnitude (0.6 to 2 points on a scale of 0–10). Notably, tinnitus was the only symptom that increased in prevalence while decreasing in severity. Some symptoms, particularly fatigue and concentration impairment, showed persistently high prevalence and high severity, while others like eczema and diarrhoea remained less common and less severe. Pairwise comparisons revealed that most significant changes in both symptom prevalence and severity occurred between baseline and second follow-up, while the interval between first and second follow-up showed little additional change, suggesting that symptom trajectories in this cohort stabilized relatively early in the observation period. A detailed examination of individual symptoms revealed that those typically associated with acute COVID-19 – such as fever, loss of smell, and loss of appetite – showed the most marked decrease in prevalence within our cohort, consistent with previous findings [22]. A longitudinal clustering analysis of Long COVID patients identified three distinct symptom patterns: (1) resolving symptoms, including runny nose, cough, and fever; (2) stable symptoms, including tachycardia, headache, nausea, and fatigue; and (3) progressive symptoms, including palpitations, tinnitus, joint pain, brain fog, and post-exertional malaise [23]. Other studies showed 4 stable clusters (no or minor symptoms; multi-symptoms; joint pain; and neurocognitive-related symptoms) with overall decreasing symptom prevalence 9 and 12 months after the initial SARS-CoV-2 infection [24]. In our cohort, fever, loss of smell, and loss of appetite showed decreasing prevalence and severity over time, while tinnitus prevalence increased. Dyspnoea also showed decreased severity. The percentage of participants with severe Long COVID according to PCS score did not change over time, because the cut-off was 1 and not the median.

In our cohort, cognitive and neuropsychiatric symptoms particularly fatigue, concentration impairment, and sleep disturbances showed high prevalence and severity that persisted over time. In accordance with our findings, a meta-analysis showed that the proportion of Long COVID patients with fatigue and cognitive impairment did not decrease over time [25], implying the potential for chronification and long-term impact on daily life. At the time of the study, no causal therapy for Long COVID was available. Given that most participants presented with symptoms relating to multiple organ systems, comprehensive multimodal treatment approaches were needed, particularly focusing on symptoms at risk of chronification, as recommended by German guidelines [26]. Our group has since investigated digital occupational therapy as a multimodal therapeutic approach for Long COVID patients, demonstrating that interactive online occupational therapy improved quality of life, cognitive function, and social participation in a randomized controlled pilot study [27, 28].

The relationship between subjective symptoms and objective findings remains complex, particularly given the fluctuating nature of Long COVID symptoms. While neuroimaging and cognitive testing have confirmed some patient-reported symptoms [29], Bungenberg et al. found cognitive test scores within normal ranges despite subjective complaints [30].

Neuropsychiatric symptoms are associated with reduced work ability, contributing to the substantial socioeconomic burden of long COVID [31]. In our previous work we have shown that Long COVID symptoms significantly impact quality of life and social participation [9, 12]. In the current analysis, we revealed that substantial impairment of social functioning persisted throughout all time points and its association did not change.

Long COVID patients report lower health-related quality of life (hrQoL) compared to the general population [32], with studies showing either stable low levels or further decreases up to two years post-infection [33]. In our cohort, hrQoL decreased over time and showed negative correlations with both symptom count and severity, despite overall improvement in mean symptom severity. A linear mixed model confirmed that symptom severity, rather than symptom count, was the primary predictor of hrQoL, accounting for within-subject correlations over time (β = −3.92, *p* < 0.001). Notably, hrQoL declined significantly over time even after controlling for symptom burden, suggesting that additional factors beyond symptom intensity may contribute to quality of life deterioration in Long COVID patients. However, the observed change of hrQoL remained below the suggested MCID of 7.5 points the smallest change perceived as meaningful by patients [21], suggesting that statistical significance should not be conflated with clinical deterioration. Nevertheless, absolute hrQoL levels remained substantially impaired relative to population norms, supporting the continued need for targeted interventions. Supporting our findings, Appel et al. also found that hrQoL decreased with increasing Long COVID severity [34]. Similar patterns can be observed in other chronic conditions, where hrQoL is reduced compared to the general population and fluctuates based on disease activity, comorbidities, coping mechanisms, and access to treatment [35, 36]. Recent longitudinal evidence confirms this heterogeneity: Schaap et al. identified distinct HrQoL trajectories in COVID-19 survivors over one year, with 44% showing persistently low physical HrQoL, underscoring that a substantial subgroup does not recover spontaneously [13]. Similarly, a Portuguese study reported persistent HrQoL impairment in more than half of Long COVID patients up to nine months after discharge, independent of disease severity or clinicodemographic characteristics [15]. It remains unclear how hrQoL evolves over the long term in individuals with Long COVID and what factors predispose to or protect against persistent impairments. Longitudinal studies can investigate these patterns to inform the development of targeted supportive interventions.

### Strengths and limitations

Our study comes with several limitations. A key limitation of our study is potential selection bias in the follow-up participation. While 3,126 participants with Long COVID completed the baseline assessment, only 262 completed all three surveys over minimum 20 weeks after the infection. This substantial dropout might reflect a selection bias toward participants with persistent symptoms, as those who recovered may have been less likely to continue participation. The broad range of time between surveys and between initial infection and baseline assessment further limits the precise comparability of timepoints; however, the consistency of findings across this range suggests robustness of the observed symptom persistence. Notably, at baseline, those who completed all surveys reported slightly more symptoms and higher symptom severity compared to those who did not complete follow-up. Therefore, our findings should be interpreted as representing the trajectory of persistent Long COVID rather than the general course of Long COVID, as we may have missed those who recovered. The online and voluntary recruitment might have caused selection bias. However, the distribution of core symptoms in our cohort aligns with those reported in a large meta-analysis [25], suggesting our sample is reasonably representative of the broader Long COVID population.

While our cohort was relatively young and predominantly female (84.4%), this demographic pattern aligns with other Long COVID studies [7, 8]. Other methodological constraints include varying intervals between assessments, and the pragmatic online recruitment that may have introduced additional selection bias. Comorbidity groupings were defined according to clinical subspecialty categories; heterogeneity within categories, particularly for neuropsychiatric and inflammatory/autoimmune conditions, may limit the interpretability of analyses. Subgroup analyses by age, gender, comorbidities, or vaccination status were beyond the scope of the present analysis but represent an important avenue for future research. While our study relied on self-reported outcomes without clinical verification (including diagnosis of SARS-CoV-2 infection), this approach aligns with the increasing recognition of patient-reported outcomes as valuable measures, particularly for conditions like Long COVID where symptoms significantly impact daily life. A further strength is the large number of participants analysed across three timepoints, providing a comprehensive longitudinal perspective. Additionally, our symptom-specific analysis not only captured the occurrence of symptoms but also their severity and impact on quality of life. Binarization of the occurrences of symptoms by median might have led to information loss, as it is a statistical value without clinical relevance. The definition of symptom presence relative to a wave-specific median is inherently arbitrary in the absence of validated thresholds, and our sensitivity analysis indicates that this choice affects the statistical power to detect changes in prevalence over time. Nonetheless, persons with different courses of COVID-19 (mild to severe) took part, enhancing the scope of our findings. Beyond longitudinal characterization, research should prioritize the identification of phenotypic subgroups to enable targeted, individualized therapeutic strategies.

## Conclusions

This longitudinal study demonstrates that among patients with persistent Long COVID, many symptoms remain challenging more than a year after infection, despite modest improvements in prevalence and severity. Participants report substantial impairments in quality of life and social participation. Given the increasing evidence that Long COVID may follow a chronic course, future interventions should prioritize accessible, home-based and digital formats alongside specialized rehabilitation programs tailored to the distinct needs of Long COVID patients, underscoring the need for individualized, multimodal treatment approaches.

## Supplementary Information

Below is the link to the electronic supplementary material.

Supplementary Material 1

## Data Availability

The datasets generated and/or analysed during the current study are not publicly available due to a decision of the responsible research ethics board but are available from the corresponding author upon reasonable request within a data sharing agreement.

## Abbreviations

CI: Confidence Interval
COVID–19: Coronavirus disease 2019
ICU: Intensive care unit
IQR: Interquartile range
MCID: Minimal clinically important difference
MHH: Hannover Medical School
NA: Not answered
OR: Odds ratio
hrQoL: Health–Related Quality of Life
PCS: Post COVID Score
SARS–CoV–2: Severe acute respiratory syndrome coronavirus type 2
UMG: University Medical Center Göttingen
