# Supplementary material for: Longitudinal observation of persistent Long COVID symptoms and health-related quality of life in an adult German cohort
Source: BMC Infect Dis. 2026 Aug 19;26:1529. doi: 10.1186/s12879-026-14252-z (PMC13491786; doi:10.1186/s12879-026-14252-z)
Supplement: Supplementary file 1 [file 12879_2026_14252_MOESM1_ESM.docx]

Supplement 1. Median symptom severity per symptom and timepoint

| **Symptom, Median (IQR)** | **Baseline** | First follow-up | **Second follow-up** |
| --- | --- | --- | --- |
| **Abdominal pain** | 3 (2-6) | 4 (2-6) | 2 (1-5) |
| **Angina pectoris** | 7 (5-8) | 5 (3-7) | 4 (3-6.5) |
| **Anxiety** | 6 (3-8) | 4 (2-7) | 4 (1-7) |
| **Arthralgia** | 7 (5-8) | 7 (5-8) | 7 (5-8) |
| **Chestpain** | 6 (4-8) | 4 (2-6) | 3 (2-6) |
| **Cough** | 4 (3-6) | 3 (1-5) | 3 (1-5) |
| **Delirium** | 4 (3-7) | 3 (1-6) | 2 (1-5) |
| **Depression** | 7 (5-8) | 7 (6-8) | 6 (4-8) |
| **Diarrhea** | 3 (1-5) | 3.5 (2-7) | 3 (1.8-5) |
| **Dyspnea** | 7 (5-8) | 6 (4.2-7) | 4 (2-6) |
| **Eczema** | 4 (2-6) | 2 (1-6) | 3 (2-6) |
| **Fatigue** | 9 (8-10) | 9 (8-10) | 8 (8-9) |
| **Fever** | 2 (1-5) | 2 (1.5-5) | 3 (1-5) |
| **Headache** | 8 (6-9) | 7 (5-8) | 6 (5-8) |
| **Hypaestaesia** | 7 (5-8) | 7 (5-8) | 7 (4-8) |
| **Loss of appetite** | 3 (1-5) | 3 (2-5) | 2 (1-5) |
| **Loss of smell** | 6.5 (2-9) | 4 (2-8) | 5 (2-8) |
| **Myalgia** | 7 (6-9) | 8 (6-9) | 7 (6-9) |
| **Nausea** | 3 (1-6) | 2.5 (1-5) | 3 (1-5) |
| **Otalgia** | 2 (1-5) | 3 (1-5) | 2 (1-4) |
| **Pain** | 8 (6-8) | 7 (6-9) | 7 (6-8) |
| **Palpitations** | 6.5 (5-8) | 6 (4-7) | 5 (3-7) |
| **Sleep disturbances** | 8 (8-10) | 8 (7.8-9) | 8 (7-9) |
| **Sore throat** | 8 (7-9) | 8 (7-9) | 7 (6-9) |
| **Tinnitus** | 4 (2-6) | 3 (2-6) | 3 (2-6) |
| **Trouble concentrating** | 4 (2-7) | 4 (2-7) | 3 (2-7) |
| **Vertigo** | 6 (4-8) | 5 (3-7) | 5 (3-7) |

Median symptom severity (scale 0–10, only among participants reporting the symptom) at baseline, first and second follow-up for all 27 Long COVID symptoms.

Supplement 2. Prevalence of Long COVID symptoms over time: omnibus and pairwise comparisons under the primary (wave-specific) and the fixed baseline cutoff.

|  | **Wave-specific cut-off** | | | | Fixed cut-off | | | |
| --- | --- | --- | --- | --- | --- | --- | --- | --- |
| **Symptom** | **P value Prevalence ^1^** | P value Baseline vs. first follow-up | **P value fist vs. second follow-up** | P value Baseline vs. second follow-up | **P value prevalance ^1^** | P value Baseline vs. first follow-up | **P value fist vs. second follow-up** | P value Baseline vs. second follow-up |
| **Abdominal pain** | 0.586 | 1.000 | 0.635 | 1.000 | 0.332 | 1.000 | 0.513 | 1.000 |
| **Angina pectoris** | 1.000 | 1.000 | 0.981 | 1.000 | <0.001 | <0.001 | 0.060 | <0.001 |
| **Anxiety** | 1.000 | 0.710 | 1.000 | 1.000 | 0.082 | 1.000 | 0.420 | 0.095 |
| **Arthralgia** | 1.000 | 1.000 | 1.000 | 1.000 | 1.000 | 1.000 | 1.000 | 1.000 |
| **Chestpain** | 1.000 | 1.000 | 1.000 | 1.000 | <0.001 | <0.001 | 0.757 | <0.001 |
| **Cough** | 1.000 | 1.000 | 1.000 | 0.691 | 0.217 | 200 | 1.000 | 0.526 |
| **Delirium** | 1.000 | 1.000 | 1.000 | 1.000 | <0.001 | 0.012 | 1.000 | <0.001 |
| **Depression** | 1.000 | 1.000 | 1.000 | 1.000 | 0.645 | 1.000 | 0.559 | 0.654 |
| **Diarrhea** | 1.000 | 1.000 | 1.000 | 1.000 | 1.000 | 1.000 | 1.000 | 1.000 |
| **Dyspnea** | 0.586 | 1.000 | 0.360 | 754 | <0.001 | 474 | 0.032 | <0.001 |
| **Eczema** | 1.000 | 1.000 | 1.000 | 1.000 | 1.000 | 1.000 | 1.000 | 1.000 |
| **Fatigue** | 1.000 | 1.000 | 1.000 | 1.000 | <0.001 | 1.000 | 0.027 | <0.001 |
| **Fever** | <0.001 | <0.001 | 1.000 | <0.001 | <0.001 | <0.001 | 1.000 | <0.001 |
| **Headache** | 1.000 | 1.000 | 1.000 | 1.000 | 0.717 | 1.000 | 1.000 | 0.457 |
| **Hypaestaesia** | 1.000 | 1.000 | 1.000 | 1.000 | 1.000 | 1.000 | 1.000 | 1.000 |
| **Loss of appetite** | <0.001 | 0.012 | 1.000 | 0.003 | <0.001 | 0.010 | 1.000 | 0.002 |
| **Loss of smell** | <0.001 | <0.001 | 0.981 | <0.001 | <0.001 | <0.001 | 0.757 | <0.001 |
| **Myalgia** | 1.000 | 1.000 | 1.000 | 1.000 | 1.000 | 1.000 | 1.000 | 1.000 |
| **Nausea** | 1.000 | 1.000 | 1.000 | 1.000 | 1.000 | 1.000 | 1.000 | 1.000 |
| **Otalgia** | 1.000 | 1.000 | 1.000 | 1.000 | 1.000 | 1.000 | 1.000 | 1.000 |
| **Pain** | 1.000 | 1.000 | 1.000 | 1.000 | 1.000 | 1.000 | 1.000 | 1.000 |
| **Palpitations** | 1.000 | 1.000 | 1.000 | 1.000 | <0.001 | 0.003 | 0.255 | <0.001 |
| **Sleep disturbances** | 1.000 | 1.000 | 1.000 | 1.000 | 1.000 | 1.000 | 1.000 | 1.000 |
| **Sore throat** | 1.000 | 1.000 | 1.000 | 1.000 | 1.000 | 1.000 | 1.000 | 1.000 |
| **Tinnitus** | <0.001 | 0.001 | 1.000 | <0.001 | <0.001 | <0.001 | 1.000 | <0.001 |
| **Trouble concentrating** | 0.612 | 0.739 | 1.000 | 0.691 | <0.001 | 0.537 | 0.044 | <0.001 |
| Vertigo | 1.000 | 1.000 | 1.000 | 1.000 | <0.001 | 0.034 | 1.000 | <0.001 |

*Prevalence across the three timepoints was compared using Cochran's Q test; pairwise contrasts using McNemar tests. P-values were adjusted for multiple testing (Bonferroni-Holm) separately within each comparison (omnibus and each pairwise contrast) across the 27 symptoms; omnibus and pairwise results may therefore differ for individual symptoms. The wave-specific cutoff was recalculated at each timepoint, the fixed cutoff was set once at the baseline median and applied unchanged across all timepoints.*

Supplement 3. Prevalence, missing values and severity of Long COVID symptoms using a cutoff fixed at the baseline median (sensitivity analysis; cf. Table 2).

|  | Baseline | | | First Follow-Up | | | Second Follow-Up | | |  |  |
| --- | --- | --- | --- | --- | --- | --- | --- | --- | --- | --- | --- |
|  | **Prevalence, n (%)** | **NA, n (%)** | **Severity, mean (SD)^1^** | **Prevalence, n (%)** | **NA, n (%)** | **Severity, mean (SD)^1^** | **Prevalence, n (%)** | **NA, n (%)** | **Severity, mean (SD)^1^** | **P value Prevalence^2^** | **p value severity^3^** |
| Abdominal pain | 107 (43.9) | 18 (6.9) | 4.13 (2.67) | 100 (38.9) | 5 (1.9) | 3.96 (2.64) | 124 (47.7) | 2 (0.8) | 3.58 (2.66) | 0.332 | 1.0 |
| Angina pectoris | 148 (58.7) | 10 (3.8) | 6.56 (1.73) | 101 (39.0) | 3 (1.1) | 6.51 (1.79) | 77 (29.4) | 0 (0.0) | 6.38 (1.65) | <0.001 | <0.001 |
| Anxiety | 126 (51.2) | 16 (6.1) | 5.75 (2.61) | 124 (48.1) | 4 (1.5) | 5.34 (2.68) | 108 (41.4) | 1 (0.4) | 5.44 (2.68) | 0.082 | 0.003 |
| Arthralgia | 132 (52.8) | 12 (4.6) | 6.81 (1.96) | 127 (49.2) | 4 (1.5) | 6.86 (1.84) | 130 (50.2) | 3 (1.1) | 6.79 (1.89) | 1.000 | 1.0 |
| Chest pain | 132 (53.2) | 14 (5.3) | 5.98 (2.10) | 98 (37.8) | 3 (1.1) | 5.65 (2.00) | 82 (31.5) | 2 (0.8) | 5.72 (1.88) | <0.001 | <0.001 |
| Cough | 130 (52.2) | 13 (5.0) | 4.67 (2.37) | 107 (41.2) | 2 (0.8) | 4.36 (2.16) | 112 (42.9) | 1 (0.4) | 4.48 (2.26) | 0.217 | 0.058 |
| Delirium | 125 (51.4) | 19 (7.3) | 4.90 (2.35) | 98 (38.4) | 7 (2.7) | 4.69 (2.29) | 91 (34.9) | 1 (0.4) | 4.29 (2.34) | <0.001 | 0.003 |
| Depression | 128 (51.2) | 12 (4.6) | 6.78 (1.80) | 133 (51.6) | 4 (1.5) | 7.08 (1.83) | 118 (45.0) | 0 (0.0) | 6.80 (2.01) | 0.645 | 0.003 |
| Diarrhea | 101 (41.1) | 16 (6.1) | 3.64 (2.66) | 88 (34.2) | 5 (1.9) | 4.12 (2.69) | 92 (35.2) | 1 (0.4) | 3.66 (2.51) | 1.000 | 1.0 |
| Dyspnea | 141 (56.6) | 13 (5.0) | 6.59 (1.66) | 123 (47.3) | 2 (0.8) | 6.29 (1.57) | 96 (36.6) | 0 (0.0) | 6.14 (1.66) | <0.001 | 0.003 |
| Eczema | 72 (30.0) | 22 (8.4) | 4.31 (2.83) | 91 (35.0) | 2 (0.8) | 3.64 (2.85) | 80 (30.8) | 2 (0.8) | 4.00 (2.60) | 1.000 | 1.0 |
| Fatigue | 170 (67.2) | 9 (3.4) | 9.02 (0.89) | 158 (61.5) | 5 (1.9) | 9.01 (0.86) | 127 (49.0) | 3 (1.1) | 8.87 (0.83) | <0.001 | 0.003 |
| Fever | 91 (37.0) | 16 (6.1) | 3.51 (2.64) | 36 (14.0) | 4 (1.5) | 3.67 (2.65) | 51 (19.5) | 1 (0.4) | 3.41 (2.42) | <0.001 | <0.001 |
| Headache | 133 (53.2) | 12 (4.6) | 7.54 (1.64) | 129 (49.8) | 3 (1.1) | 7.16 (1.62) | 120 (46.0) | 1 (0.4) | 7.07 (1.67) | 0.717 | 0.003 |
| Hypaestaesia | 136 (54.6) | 13 (5.0) | 6.74 (2.22) | 138 (53.7) | 5 (1.9) | 6.74 (2.22) | 136 (52.1) | 1 (0.4) | 6.38 (2.22) | 1.000 | 1.0 |
| Loss of appetite | 113 (46.5) | 19 (7.3) | 3.54 (2.63) | 87 (34.0) | 6 (2.3) | 3.63 (2.66) | 85 (32.7) | 2 (0.8) | 3.22 (2.56) | <0.001 | 0.003 |
| Loss of smell | 138 (55.6) | 14 (5.3) | 5.85 (3.44) | 103 (40.1) | 5 (1.9) | 5.10 (3.26) | 91 (35.0) | 2 (0.8) | 5.24 (3.21) | <0.001 | 0.003 |
| Myalgia | 137 (54.8) | 12 (4.6) | 7.55 (1.60) | 135 (52.5) | 5 (1.9) | 7.44 (1.62) | 137 (52.9) | 3 (1.1) | 7.31 (1.68) | 1.000 | 1.0 |
| Nausea | 122 (49.4) | 15 (5.7) | 3.90 (2.79) | 117 (45.3) | 4 (1.5) | 3.48 (2.52) | 114 (43.5) | 0 (0.0) | 3.40 (2.55) | 1.000 | 0.606 |
| Otalgia | 69 (28.2) | 17 (6.5) | 3.45 (2.81) | 59 (22.8) | 3 (1.1) | 3.54 (2.55) | 73 (28.0) | 1 (0.4) | 3.08 (2.20) | 1.000 | 1.0 |
| Pain | 144 (57.6) | 12 (4.6) | 7.35 (1.64) | 142 (55.3) | 5 (1.9) | 7.31 (1.62) | 146 (55.9) | 1 (0.4) | 7.32 (1.60) | 1.000 | 1.0 |
| Palpitations | 154 (61.6) | 12 (4.6) | 6.58 (1.95) | 125 (48.1) | 2 (0.8) | 6.22 (1.71) | 102 (39.1) | 1 (0.4) | 6.41 (1.70) | <0.001 | 1.0 |
| Sleep disturbances | 136 (55.3) | 16 (6.1) | 8.15 (1.37) | 128 (49.6) | 4 (1.5) | 8.07 (1.38) | 125 (47.9) | 1 (0.4) | 8.14 (1.41) | 1.000 | <0.001 |
| Sore throat | 137 (55.7) | 16 (6.1) | 4.03 (2.63) | 150 (57.9) | 3 (1.1) | 3.87 (2.51) | 159 (60.9) | 1 (0.4) | 4.12 (2.67) | 1.000 | 0.208 |
| Tinnitus | 102 (42.5) | 22 (8.4) | 4.67 (3.08) | 140 (54.3) | 4 (1.5) | 4.75 (3.02) | 157 (60.4) | 2 (0.8) | 4.29 (3.07) | <0.001 | 0.003 |
| Trouble concentrating | 155 (61.0) | 8 (3.1) | 8.55 (1.12) | 139 (53.9) | 4 (1.5) | 8.28 (1.01) | 114 (43.7) | 1 (0.4) | 8.32 (0.97) | <0.001 | <0.001 |
| Vertigo | 148 (59.2) | 12 (4.6) | 5.99 (2.25) | 125 (48.4) | 4 (1.5) | 5.97 (2.20) | 115 (44.1) | 1 (0.4) | 5.70 (1.99) | <0.001 | <0.001 |

*Prevalence, n (%) of participants classified as symptomatic; NA, n (%) of missing ratings; severity, mean (SD) among symptomatic participants. ²P value prevalence: Cochran's Q test. ³P value severity: Skillings-Mack test. Both Bonferroni-Holm adjusted across the 27 symptoms. Severity p-values are identical to Table 2, as the severity test uses the raw 0–10 ratings of all participants and is therefore independent of the presence threshold.*

Supplement 4. P-values of post-hoc pairwise comparisons of symptom severity across three time points

| Symptom | P value intensity | P value Baseline vs. first follow-up | P value first vs. second follow-up | P value Baseline vs. second follow-up |
| --- | --- | --- | --- | --- |
| **Abdominal pain** | 1.0 | 1.0 | 1.0 | 1.0 |
| **Angina pectoris** | <0.001 | <0.001 | 0.016 | <0.001 |
| **Anxiety** | 0.003 | 0.811 | 0.548 | 0.002 |
| **Arthralgia** | 1.0 | 1.0 | 1.0 | 1.0 |
| **Chestpain** | <0.001 | <0.001 | 0.997 | <0.001 |
| **Cough** | 0.058 | 0.060 | 1.0 | 0.608 |
| **Delirium** | 0.003 | <0.001 | 0.331 | <0.001 |
| **Depression** | 0.003 | 1.0 | 0.002 | 0.182 |
| **Diarrhea** | 1.0 | 1.0 | 1.0 | 1.0 |
| **Dyspnea** | 0.003 | 0.005 | 0.005 | <0.001 |
| **Eczema** | 1.0 | 1.0 | 1.0 | 1.0 |
| **Fatigue** | 0.003 | 0.634 | <0.001 | <0.001 |
| **Fever** | <0.001 | <0.001 | 1.0 | 0.014 |
| **Headache** | 0.003 | 0.012 | 1.0 | 0.001 |
| **Hypaestaesia** | 1.0 | 1.0 | 1.0 | 1.0 |
| **Loss of appetite** | 0.003 | 0.333 | 1.0 | 0.019 |
| **Loss of smell** | 0.003 | <0.001 | 0.844 | <0.001 |
| **Myalgia** | 1.0 | 1.0 | 1.0 | 1.0 |
| **Nausea** | 0.606 | 1.0 | 1.0 | 0.250 |
| **Otalgia** | 1.0 | 1.0 | 1.0 | 1.0 |
| **Pain** | 1.0 | 1.0 | 1.0 | 1.0 |
| **Palpitations** | 1.0 | <0.001 | 1.0 | <0.001 |
| **Sleep disturbances** | <0.001 | 0.405 | 1.0 | 0.278 |
| **Sore throat** | 0.208 | 1.0 | 1.0 | 1.0 |
| **Tinnitus** | 0.003 | <0.001 | 1.0 | 0.001 |
| **Trouble concentrating** | <0.001 | 0.001 | 0.043 | <0.001 |
| **Vertigo** | <0.001 | 0.018 | 0.129 | <0.001 |

*Omnibus comparisons were performed using the Skillings-Mack test; pairwise comparisons using Wilcoxon signed-rank tests. All severity scores (rated 0–10) from all participants with a valid rating at the respective timepoints were included. P-values were adjusted for multiple testing using the Bonferroni-Holm method.*

Supplement 5. Linear mixed model for the association between symptom burden and health-related quality of life

| **Predictor** | **β** | **SE** | **95% CI** | **t** | **df** | **p** |
| --- | --- | --- | --- | --- | --- | --- |
| Symptom count | -0.19 | 0.19 | -0.57; 0.20 | -0.95 | 753.96 | 0.341 |
| Symptom severity | -3.92 | 0.72 | -5.32; -2.52 | -5.46 | 752.96 | <0.001 |
| Time: first follow-up vs baseline | -3.51 | 1.62 | -6.68; -0.33 | -2.16 | 626.89 | 0.031 |
| Time: second follow-up vs baseline | -6.34 | 1.72 | -9.71; -2.97 | -3.68 | 646.82 | <0.001 |

*Results of a linear mixed model with EQ5D-VAS as outcome, symptom count, mean symptom severity, and timepoint as fixed effects, and a random intercept per participant (n = 262). β = unstandardized regression coefficient; SE = standard error; 95% CI = confidence interval; df = degrees of freedom. Reference category for time is baseline.*
